# Respiratory Virus Prevalence Across Pre-, During-, and Post-SARS-CoV-2 Pandemic Periods

**DOI:** 10.3390/v17081040

**Published:** 2025-07-25

**Authors:** Michele Manno, Grazia Pavia, Simona Gigliotti, Marta Pantanella, Giorgio Settimo Barreca, Cinzia Peronace, Luigia Gallo, Francesca Trimboli, Elena Colosimo, Angelo Giuseppe Lamberti, Nadia Marascio, Giovanni Matera, Angela Quirino

**Affiliations:** Unit of Clinical Microbiology, Department of Health Sciences, “Magna Græcia” University, A.O.U. “R. Dulbecco”, 88100 Catanzaro, Italy; michele.manno@unicz.it (M.M.); graziapavia@unicz.it (G.P.); s.gigliotti@unicz.it (S.G.); marta.pantanella@studenti.unicz.it (M.P.); gbarreca@unicz.it (G.S.B.); cinzia.peronace@aourenatodulbecco.it (C.P.); trimboli@unicz.it (F.T.); alambert@unicz.it (A.G.L.); mmatera@unicz.it (G.M.); quirino@unicz.it (A.Q.)

**Keywords:** prevalence of viral respiratory infections, COVID-19 pandemic, SARS-CoV-2

## Abstract

The COVID-19 pandemic significantly impacted the circulation, seasonality, and disease burden of viral respiratory infections. This study aimed to evaluate the impact of SARS-CoV-2 on the frequency of viral respiratory infections at a teaching hospital in Southern Italy by comparing data from before, during, and after the COVID-19 pandemic and by investigating how the emergence of SARS-CoV-2 affected the circulation and seasonality of other respiratory viruses. This retrospective and prospective study was performed on de-identified nasopharyngeal specimens classified as pre-COVID-19 (before 15 March 2020), during-COVID-19 (from 16 March 2020 to 5 May 2023), and post-COVID-19 (from 6 May 2023 to 31 December 2024). Overall, 790 out of 3930 (20%) patient samples tested positive for at least one respiratory virus. The mean age of patients was 60 ± 19 years, with significant positivity rates observed in the 65–98 age group (*p* ≤ 0.05) across all periods. In the pre-COVID-19 period, the most prevalent virus was influenza A (47.5%, 47/99), followed by the human rhinovirus (19.2%, 19/99). During the COVID-19 pandemic, SARS-CoV-2 was the most prevalent (64.9%, 290/447), before decreasing to 38% (92/244) after the pandemic, while influenza A’s positivity prevalence increased to 14.3% (35/244). Rhinovirus/enterovirus remained relatively stable throughout all periods. The pandemic notably altered viral co-infection dynamics, with its effects lasting into the post-COVID-19 period. Specifically, a marked decrease in influenza A circulation was observed, while respiratory syncytial virus (RSV) epidemiology remained stable and significant co-circulation of rhinovirus/enterovirus with SARS-CoV-2 persisted. Therefore, since COVID-19 and influenza affect the same high-risk groups, those individuals must be vaccinated against both viruses.

## 1. Introduction

Viral respiratory tract infections (vRTIs) include a broad spectrum of diseases, ranging from asymptomatic to severe, life-threatening conditions, with heightened morbidity and mortality in young children, the elderly, and immunocompromised populations [1]. In recent years, more than 200 different virus families have been identified as causes of viral acute respiratory infections (vARIs) in both the upper and lower respiratory tracts [2]. Some of these, originating from animals, can adapt to infect humans and spread quickly through society, particularly affecting immunocompromised individuals, resulting in epidemics or pandemics [3,4,5,6,7]. Common pathogens responsible for vARIs include adenovirus (AdV), enterovirus (EV), coronavirus (CoV), human metapneumovirus (HMPV), rhinovirus (RV), influenza and parainfluenza viruses, human bocavirus (HBoV), and respiratory syncytial virus (RSV) [8,9].

The onset of the COVID-19 pandemic in late 2019 led to unprecedented public health measures that drastically altered the epidemiology of viral respiratory pathogens [10]. A viral pandemic, similar to SARS-CoV-2 in its pathogenesis, histopathology, worldwide elevated mortality, rate of spread, and frequent coinfection with pulmonary pathogens was the 1918 influenza pandemic (“Spanish” influenza) caused by the influenza A (H1N1) virus [11]. Before the COVID-19 pandemic, seasonal influenza and RSV were leading contributors to morbidity and mortality, with established patterns of circulation mainly among infants and older adults [12,13]. During the pandemic, a wide range of stringent non-pharmaceutical interventions (NPIs), including maintaining physical distance, wearing face masks, limiting travel, shutting down schools and businesses, and promoting hand hygiene, effectively reduced SARS-CoV-2 transmission but notably influenced the prevalence and epidemiology of other respiratory pathogens [8,14,15]. In particular, reducing exposure to common viral pathogens may lead to an “immunity debt”, which could increase the susceptibility to future vRTIs [16,17]. The shift of SARS-CoV-2 from a pandemic to an endemic phase has created a complex environment for viral co-circulation, encompassing not only various SARS-CoV-2 variants but also other respiratory pathogens such as HRV, influenza, AdV, RSV, and seasonal endemic coronaviruses. Co-infections involving SARS-CoV-2 and other respiratory viruses may alter the clinical presentation of the disease, which complicates diagnosis and treatment [18,19,20,21]. Recent advances in multiplex PCR platforms have enabled the simultaneous identification of multiple respiratory viruses and bacteria, thus offering their rapid and efficient detection in patient specimens [22]. Rapid molecular testing has notably improved detection by allowing simultaneous identification of multiple pathogens, enhancing sensitivity and specificity, reducing time to positivity, and ultimately expediting clinical treatment [22].

Herein, we report the prevalence of respiratory viruses in hospitalized patients attending a teaching hospital in Southern Italy, comparing pre-, during-, and post-COVID-19 pandemic periods. Specifically, we focused on how the emergence of SARS-CoV-2 influenced the circulation of other respiratory viruses, such as influenza viruses, HRV, and RSV, as well as how pandemic-related disruptions in healthcare and viral transmission patterns affected their prevalence during and after the pandemic.

## 2. Materials and Methods

### 2.1. Study Design

**Figure 1:**
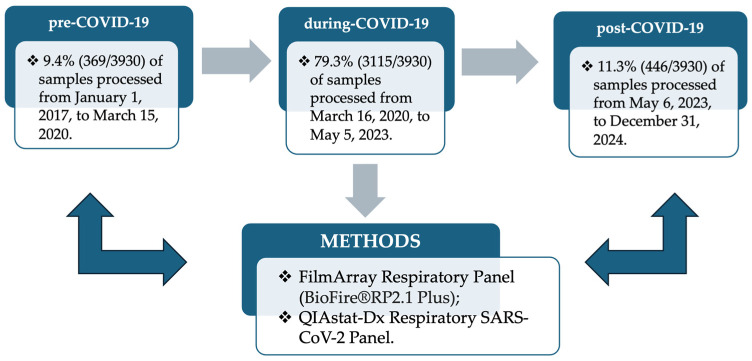
Flowchart of study design.

In this study, we analyzed nasopharyngeal swabs (NPSs) that had been retrospectively and prospectively collected from hospitalized patients with suspected vARIs attending the “Renato Dulbecco” University Hospital of Catanzaro (Italy) from January 2017 to December 2024. The NPSs were de-identified by removing all personal identifiers, ensuring patient privacy and allowing their use for research purposes without the possibility of tracing data back to individual subjects. The processed respiratory samples were classified into pre-COVID-19, during-COVID-19, and post-COVID-19, and the analysis focused exclusively on respiratory viruses, excluding bacterial pathogens, as illustrated in Figure 1. The date 5 May 2023 was chosen as the start of the post-COVID-19 pandemic period as it was the day on which the World Health Organization (WHO) officially declared an end to the COVID-19 pandemic as a global health emergency. For each period, the following data were evaluated: the prevalence of viral respiratory pathogens, the age-related stratification of vARIs (0–17; 18–64; 65–98-year ranges), and the circulation of viral agents across different hospital wards, such as the Infectious Diseases Unit (IDU), the ICU, other hospital departments (Cardiology, Urology, Oncology, Respiratory Medicine, Pediatrics, Orthopedics, Metabolic Diseases, Ophthalmology, Gastroenterology, Hepatology, Nephrology, Neurology, Internal Medicine, Forensic and Legal Medicine, and surgical specialties), triage, and outer patients. Additionally, viral co-infection patterns during all three periods were evaluated. Descriptive statistical analysis was conducted, including data such as demographic characteristics and the prevalence of viral respiratory pathogens.

### 2.2. Multiplex Real-Time PCR Assays for the Diagnosis of vARIs

NPS samples were processed according to the manufacturer’s protocols. The FilmArray RP2.1 Plus (bioMérieux Italia S.p.A., Florence, Italy) is a fully automated multiplex PCR assay designed to detect 17 viral respiratory pathogens and four bacterial species (Supplementary Table S1) [23]. The QIAstat-Dx Respiratory SARS-CoV-2 Panel (QIAGEN^®^ S.r.l, Milan, Italy) is a qualitative molecular test performed using the QIAstat-Dx Analyzer 1.0 (QIAGEN^®^ S.r.l, Milan, Italy), which integrates nucleic acid extraction with real-time multiplex PCR detection (Supplementary Table S1) [24].

In the pre-pandemic period, all specimens were analyzed using the FilmArray RP2.1 Plus (bioMérieux Italia S.p.A., Florence, Italy), a multiplex PCR system for the detection of common respiratory pathogens. Following the onset of the COVID-19 pandemic and in response to the increased diagnostic demand, the QIAstat-Dx Respiratory SARS-CoV-2 Panel (QIAGEN^®^ S.r.l, Milan, Italy) was additionally implemented to expand testing capacity and ensure timely processing of the high volume of clinical samples [24]. The biological samples that had been gathered during the acute symptomatic phase were collected using swabs from the Copan Universal Transport Medium (UTM) system. The collected material was stored at +4 °C until its delivery to the laboratory. An aliquot (300 μL) of each prepared sample was used for molecular biology analyses, while the remaining part was preserved at −80 °C.

## 3. Results

### 3.1. Demographic Characteristics of Patient Samples for vARI Diagnosis

A total of 3930 patients’ NPSs were analyzed, with a gender distribution of 1986 males (50.5%) and 1944 females (49.5%). The median age of the patients was 60 years, with a standard deviation of ±19. Out of the total, 790 samples (20%, 790/3930) tested positive for respiratory viruses, including 473 males (60%) and 317 females (40%). The positive samples were also classified as belonging to three different time periods: 12.5% (99/790) were collected during the pre-COVID-19 era, 56.6% (447/790) during the COVID-19 pandemic, and 30.9% (244/790) during the post-COVID-19 period. Positive NPS samples were predominantly obtained from the Infectious Diseases Unit (IDU), with 349 samples (44.2%, 349/790). Other sources included various hospital departments* (24.8%, 196/790), the Intensive Care Unit (ICU) (18.9%, 149/790), triage areas (10.4%, 82/790), and outpatient settings (1.9%, 15/790) (Table 1).

### 3.2. The Prevalence of Respiratory Pathogens in the Pre-, During-, and Post-COVID-19 Era

**Figure 2:**
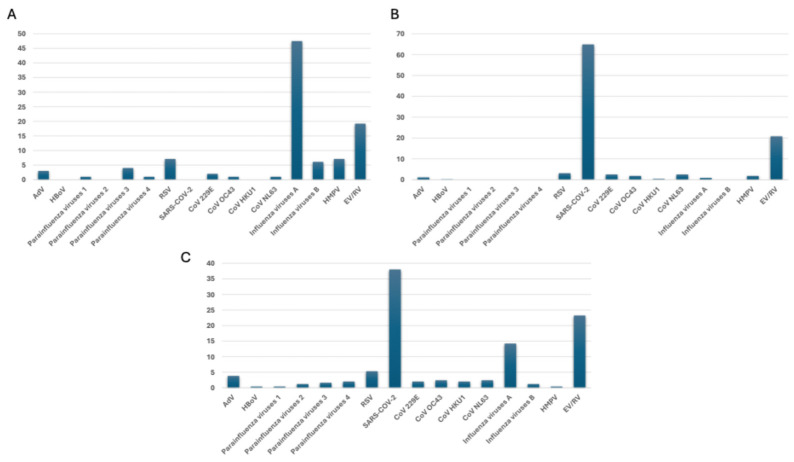
The prevalence of respiratory pathogens (**A**) pre-, (**B**) during-, and (**C**) post-COVID-19 pandemic, as evaluated by multiplex real-time PCR assays.

In the pre-COVID-19 era, influenza A was the most common virus, being detected in 47.5% (47/99) of respiratory samples. EV/RV was the second most frequent, accounting for 19.2% (19/99) of cases. RSV and HMPV were also notable, each detected in 7.1% (7/99) of cases, particularly affecting pediatric populations. Among parainfluenza viruses, parainfluenza 3 was the most frequently detected strain (4%, 4/99), while other types, including parainfluenza 1 (1%, 1/99) and parainfluenza 4 (1%, 1/99), were found less often. Among coronaviruses, strains 229E (2%, 2/99), OC43 (1%, 1/99), and NL63 (1%, 1/99) were identified, though their prevalence was far lower compared to Influenza A and EV/RV (Figure 2A). During the COVID-19 pandemic, the circulation of several respiratory viruses was significantly impacted. Influenza A experienced a dramatic decline, plummeting to just 0.9% (4/447), while influenza B was completely absent. RSV also saw a substantial decrease, dropping to 3.1% (14/447). Parainfluenza viruses 1, 3, and 4 disappeared entirely, and parainfluenza virus 2 remained undetected throughout the pandemic. In contrast, SARS-CoV-2 emerged as the predominant pathogen, accounting for 64.9% (290/447) of cases. Additionally, a slight increase was observed in EV/RV (20.8%, 93/447) and some seasonal coronaviruses, particularly NL63 (2.5%, 11/447) and OC43 (1.8%, 8/447) (Figure 2B). During the post-COVID-19 pandemic period, SARS-CoV-2 kept its place as the dominant pathogen, accounting for 38% (92/244) of cases. EV/RV remained prevalent at 23.3% (56/244), while RSV was present in 5.3% (13/244) of cases. The prevalence of influenza viruses significantly decreased, with influenza A detected in 14.3% (35/244) of cases and influenza B in 1.2% (3/244), suggesting reduced circulation due to pandemic-related measures. Coronaviruses such as 229E, OC43, HKU1, and NL63 continued circulating but at lower frequencies (ranging from 2% to 2.4%). Other viruses, including AdV (3.7%, 9/244) and HMPV (0.4%, 1/244), were detected at relatively low levels. The parainfluenza viruses showed low prevalence, with parainfluenza 3 being the most common at 1.6% (4/244) (Figure 2C).

### 3.3. Age-Related Stratification of Respiratory Pathogens in the Pre-, During-, and Post-COVID-19 Era

**Figure 3:**
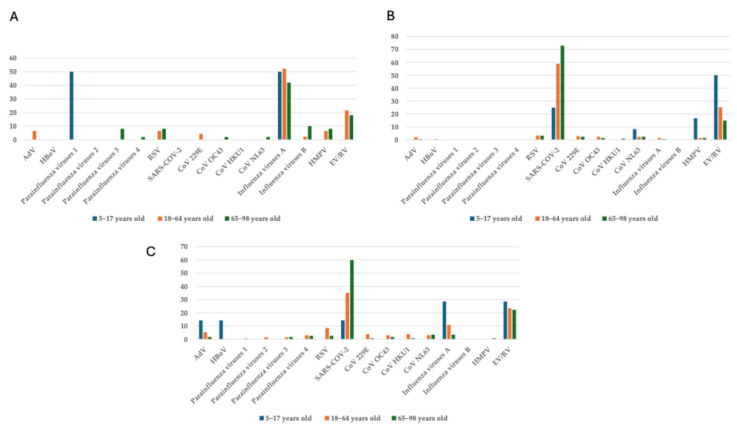
Age-related stratification (5–17; 18–64; 65–98-year ranges) of vARIs in the (**A**) pre-, (**B**) during-, and (**C**) post-COVID-19 periods.

The prevalence of respiratory viruses also varied across age groups. Among children and adolescents (5–17 years), parainfluenza 1 and influenza A were most prevalent, each at 50% (1/2, respectively). In adults (18–64 years), influenza A remained dominant (52.2%, 24/46), followed by EV/RV (21.7%, 10/46), with RSV and HMPV detected in 6.5% (3/46) of cases each. In the elderly (65–98 years), influenza A continued to be the most common (42%, 21/51), with RSV and HMPV at 8% (4/51, respectively) each and EV/RV at 18% (9/51) (Figure 3A). The prevalence of SARS-CoV-2 also varied significantly across different age groups. In the pediatric setting, 25% (3/12) of individuals were infected with SARS-CoV-2, whereas considerably higher incidences were observed in adults (58.9%, 126/214) and elderly individuals (72.9%, 161/221). EV/RV remained prevalent, especially in the child and adolescent group (50%, 6/12) and in the adult group (25.2%, 54/214); RSV, on the other hand, was not detected in children and adolescents, and it only affected 3.3% of adults (7/214) (Figure 3B). As to age-related patterns, notable changes in the distribution of respiratory viruses were observed in the post-pandemic period. SARS-CoV-2 remained the most prevalent virus across all age groups, particularly among individuals aged 65–98, wherein it accounted for 59.8% (66/112) of cases. In the children and adolescents’ group (5–17 years), EV/RV and influenza virus A showed the highest prevalence (28.6%, 2/7, respectively), while SARS-CoV-2, HBoV, and AdV were each detected in 14.3% (1/7, respectively) of individuals. In the adult age group (18–64 years), SARS-CoV-2 was the most prevalent (33.6%, 42/125), followed by EV/RV (22.4%, 28/125) and by influenza A (10.4%, 13/125). Notably, Influenza A circulation declined across all age groups, with its lowest prevalence (2.6%, 3/112) observed in the elderly. RSV and parainfluenza viruses were detected only sporadically (2.6%, 3/112, respectively) across all age groups (Figure 3C).

### 3.4. Viral Co-Infection Patterns During Pre-, During-, and Post-COVID-19 Pandemic Periods

Before the COVID-19 pandemic, viral co-infections were observed, with certain combinations being more frequently reported. Notably, co-infections involving influenza A, influenza B, HMPV, EV/RV, and AdV were detected in 16.6% of cases. Other combinations, such as EV/RV with RSV or coronaviruses (NL63, 229E), also occurred at similar rates. However, many co-infections, including those involving SARS-CoV-2, were absent, as the virus had not yet emerged. The prevalence of viral coinfections increased substantially during the COVID-19 pandemic, with a total of 38 cases observed. This significant rise was largely driven by SARS-CoV-2, which frequently co-infects along with other respiratory viruses. The most prevalent combinations involved SARS-CoV-2 alongside EV/RV (34.2%), RSV (10.5%), and various other coronaviruses. In the post-COVID-19 period, a total of 10 viral co-infections were observed. The most significant co-infections involved SARS-CoV-2, including combinations with EV/RV (20%), influenza A (20%), and coronavirus NL63 (10%). Additionally, co-infections with EV/RV and RSV were present in 10% of cases, as well as other combinations, such as influenza A with CoV-229E, AdV with HBoV, and parainfluenza 3 with EV/RV, each found in 10% of cases. Notably, co-infections involving HMPV, influenza B, and parainfluenza viruses were absent in the post-COVID-19 period (Table 2).

### 3.5. Impact of COVID-19 on the Seasonal Dynamics and Detection Rates of Major Respiratory Viruses (2017–2024)

**Figure 4:**
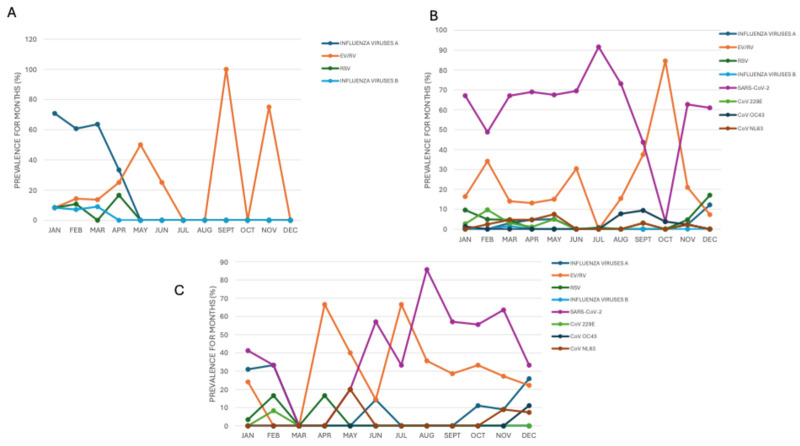
Seasonality trend of circulating respiratory viruses in the (**A**) pre-, (**B**) during- and (**C**) post-pandemic COVID-19 period.

The COVID-19 pandemic greatly altered usual seasonal patterns of major respiratory viruses. Influenza A, which normally peaks sharply in winter months (January: 70.8%, 17/24; February: 60.7%, 17/28) and in March (63.6%, 14/22), experienced a significant decline during the pandemic, with its circulation largely slowing down and much lower detection rates, even during peak months. After the pandemic, influenza A partially regained its winter seasonality (February 33.3%, 4/12), December 25.9%, 7/27) but displayed unusual activity, including unexpected detections in summer (June 14.3%, 1/7), indicating a shift from its traditional pattern. Similarly, RSV faced a steep decline during the pandemic, nearly disappearing except for minor peaks in January (9.6%, 7/73) and December (17.1%, 7/41), a noticeable change from its typical prevalence in late winter and spring (February 10.7%, 3/28, April 16.6%, 2/12). RSV’s return after the pandemic was delayed and less intense, which indicated persistent disruption of its pattern. In contrast, EV/RV proved highly resilient to COVID-19 control measures, maintaining high levels throughout the year both during and after the pandemic. Its seasonal peaks became less distinct, with significant detection during typically low-circulation periods such as summer (April and July, both at 66.6%, 4/6) and fall (October 33.3%, 3/9), a demonstration of its ability to endure despite interventions. Additionally, circulation of other seasonal human coronaviruses (HCoV 229E, NL63, OC43) was greatly reduced or altered during the pandemic, with only sporadic low-level detections of CoV-229E, peaking at 9.7% (4/41) in February (Figure 4).

## 4. Discussion

### 4.1. Study Overview and Diagnostic Approach

In this retrospective and prospective study, we analyzed the prevalence of viral respiratory infections from 2017 to 2024 using multiplex real-time PCR assays. These panels for detecting respiratory viruses have been highlighted as a primary diagnostic approach for identifying nucleic acids from viral or bacterial organisms in respiratory tract infections. These tools are crucial for detecting vARIs due to the potential presence of organisms in clinical samples at low levels, which decrease rapidly over time despite ongoing symptoms. Our study’s findings show the distribution of various pathogens among patients presenting with respiratory infection symptoms at a teaching hospital in southern Italy. Additionally, this research offers new insights into the prevalence of viral respiratory infections before, during, and after the COVID-19 pandemic.

### 4.2. Impact of the COVID-19 Pandemic on Respiratory Virus Epidemiology

Multiple studies have demonstrated that patients with respiratory tract diseases often have various viruses detected, with reported frequencies as high as 35% [25,26]. Our study demonstrates that the COVID-19 pandemic profoundly impacted respiratory virus prevalence. During the pandemic, viruses such as adenovirus (AdV) and respiratory syncytial virus (RSV) decreased due to public health measures like social distancing and mask usage but rebounded or even exceeded pre-pandemic levels afterward. Human bocavirus (HBoV) emerged during the pandemic and increased post-pandemic, indicating shifts in viral transmission dynamics. Influenza A and parainfluenza viruses sharply declined during the pandemic but partially resurged after the pandemic, in line with the temporary disruption of their circulation. Consistently with another research, EV/RV remained highly prevalent throughout, slightly increasing from 19.2% before the pandemic to 23.2% after the pandemic, underscoring its role as a dominant respiratory pathogen [27,28,29,30].

### 4.3. Gender and Age-Related Differences in Viral Prevalence

Gender differences were observed: AdV and SARS-CoV-2 were more common in males, with parainfluenza 3 re-emerging chiefly in males after the pandemic. Coronaviruses OC43 and NL63 showed gender-specific trends during the pandemic, affecting males and females differently. Such variations suggest that biological, behavioral, and healthcare access factors may influence virus susceptibility, emphasizing the importance of gender-specific public health strategies. Age-related patterns revealed that SARS-CoV-2 predominantly affected elderly individuals during the pandemic. In the aftermath of the pandemic, its prevalence decreased, while AdV and HBoV infections increased in children. Influenza A and EV/RV remained significant across all age groups, highlighting the need for age-targeted preventive measures, particularly for vulnerable populations during pandemic events [31].

### 4.4. Impact of COVID-19 on the Seasonality of Respiratory Viruses

The COVID-19 pandemic significantly altered the seasonal circulation of major respiratory viruses. Influenza A and RSV, which previously showed well-defined peaks in winter and early spring, experienced a marked decline during the pandemic and only partially recovered their typical seasonality in the post-pandemic period, while still showing some unusual off-season activity (e.g., influenza A in June). In contrast, EV/RV remained consistently active throughout all periods, showing resistance to control measures and a lack of strict seasonal behavior. Other seasonal coronaviruses (HCoVs) showed minimal circulation, likely due to mitigation strategies and competition with SARS-CoV-2. Overall, the pandemic has led to lasting shifts in respiratory virus ecology, with altered patterns that persist beyond the height of the pandemic [10,12,13].

### 4.5. Implications of Reduced Population Immunity and Environmental Factors

The interruption of respiratory virus circulation during the pandemic likely reduced herd immunity, potentially increasing susceptibility to more severe infections in the post-pandemic period [32]. This effect may have mitigated environmental influences such as high temperatures that usually suppress off-season viral activity [16,33].

### 4.6. Dynamics of Viral Coinfections During and After the Pandemic

Viral coinfections significantly increased during the COVID-19 pandemic, largely driven by SARS-CoV-2 co-infections with EV/RV, RSV, and other coronaviruses. While coinfections decreased after the pandemic, rates remained above pre-pandemic levels, suggesting lasting changes in viral interaction dynamics. Clinical studies have associated SARS-CoV-2 coinfections with more severe disease manifestations, particularly when combined with rhinovirus or enterovirus infections [34,35,36,37]. The resurgence of seasonal respiratory viruses, such as influenza A virus, and the continued circulation of SARS-CoV-2 warrant the focus on understanding how respiratory viruses interact within the host. Even if one might intuitively expect co-infections to exacerbate disease, growing evidence suggests that viral interference, in which infection with one virus decreases the replication of the other, may also occur. Recent studies suggest that SARS-CoV-2 may inhibit co-circulating respiratory viruses through innate immune-mediated mechanisms, including the early induction of interferon-stimulated genes, which reduces susceptibility to secondary viral infections [38,39,40,41]. This negative viral interaction, particularly between SARS-CoV-2 and influenza A virus, could partly explain the marked drop in influenza A prevalence during the pandemic and the limited number of co-infections detected in our study. Experimental and epidemiological data further support the concept that viral competition may alter both the timing and intensity of epidemic waves, leading to temporary suppression of one virus in the presence of another with higher transmissibility or replication advantage [38,39,40,41]. Therefore, our findings are consistent with this emerging evidence and warrant further investigation into the immunological and ecological mechanisms underlying dynamics interaction in viral co-circulation scenarios. Beside the aforementioned viral coinfection, it may be of interest that mixed viral infections have been recently found to have taken place during other pandemic events, such as the 1918 Spanish flu [11].

### 4.7. Performance and Limitations of Multiplex PCR Diagnostic Tools and Clinical Interpretation Challenges

Our study supports the reliability of multiplex PCR assays such as FilmArray RP 2.1 and the QIAstat Respiratory Panel in accurately detecting a broad range of respiratory viruses, including SARS-CoV-2, demonstrating excellent diagnostic performance consistent with previous evaluations [42]. These rapid, multiplex platforms facilitate the timely identification of pathogens, improving clinical decision-making and enabling better antimicrobial stewardship. However, confirmatory testing using quantitative RT-PCR assays is recommended for positive SARS-CoV-2 results obtained via FilmArray to ensure diagnostic accuracy.

However, it must be noted that this study lacks clinical data, which would usually be able to interpret coinfections detected by multiplex panels correctly. This limitation underscores the need for cautious interpretation of multiplex assay results in clinical practice, ideally integrating clinical presentation and additional laboratory findings. Despite these challenges, our findings contribute valuable epidemiological insights into respiratory viral coinfections and underscore the usefulness and limitations of multiplex diagnostics in real-world hospital settings.

## 5. Conclusions

In summary, the COVID-19 pandemic significantly altered the epidemiology of respiratory viruses during and after the pandemic. Before the pandemic, influenza A and EV/RV were predominant. During the pandemic, SARS-CoV-2 became the dominant pathogen, while many seasonal viruses—including influenza and parainfluenza—virtually disappeared. In the post-pandemic phase, SARS-CoV-2 remained prevalent, particularly among older adults, while EV/RV, RSV, and influenza A showed partial resurgence with altered age-specific distributions. These shifts highlight the importance of continuous, multi-pathogen surveillance to promptly detect epidemiological changes. In this respect, our virological data from January to May 2025 were included in the regional as well as the national epidemiological and virological surveillance system for influenza and other respiratory viruses (RespiVirNet) supervised by the Istituto Superiore di Sanità (ISS), Rome, Italy. In this time span, we detected 33.4% (67/201) of positive samples among patients with suspected vARIs.

The use of multiplex PCR platforms like FilmArray RP 2.1 Plus and QIAstat enhances diagnostic accuracy, optimizes patient management, and supports antimicrobial stewardship. To mitigate future respiratory outbreaks, health systems must prioritize integrated surveillance, targeted vaccination campaigns for high-risk groups, and dynamic response systems capable of adjusting to changing viral circulation patterns. The post-pandemic landscape offers a unique opportunity to strengthen public health resilience through proactive, data-driven approaches.

## Figures and Tables

**Table 1:** Patient cohort (N = 3930) evaluated for suspected vARIs during 2017–2024.

	Pre-COVID-19 (N = 369)	During-COVID-19 (N = 3115)	Post-COVID-19 (N = 446)
NPS positive samples for period% (n/N)	26.8 (99/369)	14.3 (447/3115)	54.7 (244/446)
NPS samples by age group% (n/N)			
age 5–17 years	2 (2/99)	2.7 (12/447)	2.8 (7/244)
age 18–64 years	46.4 (46/99)	47.8 (214/447)	51.2 (125/244)
age 65–98 years	51.5 (51/99)	49.4 (221/447)	45.9 (112/244)
Number of samples positive for season% (n/N)			
Fall (September–November)	5 (5/99)	19.4 (87/447)	14.7 (36/244)
Spring (March–May)	36.4 (36/99)	36.6 (164/447)	13.5 (33/244)
Summer (June–August)	5 (5/99)	9.17 (41/447)	26.2 (64/244)
Winter (December–February)	53 (53/99)	34.67 (155/447)	45.4 (111/244)
Ward of viral isolation% (n/N)			
IDU	10.1 (10/99)	58.2 (260/447)	32.3 (79/244)
Other hospital wards *	27.3 (27/99)	17.4 (78/447)	37.3 (91/244)
ICU	52.6 (52/99)	8.3 (37/447)	24.6 (60/244)
Triage	1.0 (1/99)	15.2 (68/447)	5.3 (13/244)
Outpatient	8.0 (8/99)	0.9 (4/447)	1.2 (3/244)

NPS: nasopharyngeal swab; IDU: Infectious Diseases Unit; ICU: Intensive Care Unit. * Cardiology, Urology, Oncology, Respiratory Medicine, Pediatrics, Orthopedics, Metabolic Diseases, Ophthalmology, Gastroenterology, Hepatology, Nephrology, Neurology, Internal Medicine, Forensic and Legal Medicine, and surgical specialties.

**Table 2:** Viral coinfection patterns in pre-, during-, and post-COVID-19 pandemic periods.

Coinfection	Pre-COVID-19 N (%)	During-COVID-19 N (%)	Post-COVID-19 N (%)
HMPV + Influenza B	1 (16,6)	0	0
Influenza A + Influenza B	1 (16,6)	0	0
EV/RV + RSV	1 (16,6)	1 (2,6)	1 (10)
EV/RV + AdV	1 (16,6)	3 (7,9)	0
EV/RV + CoV 229E	0	1 (2,6)	0
EV/RV + CoV NL63	1 (16,6)	0	0
Influenza A + AdV	1 (16,6)	0	0
EV/RV + SARS-CoV-2	0	13 (34,2)	2 (20)
RSV + SARS-CoV-2	0	4 (10,5)	0
CoV NL63 + SARS-CoV-2	0	1 (2,6)	0
CoV OC43 + SARS-CoV-2	0	2 (5,2)	0
CoV 229E + SARS-CoV-2	0	4 (10,5)	0
CoV HKU1 + SARS-CoV-2	0	1 (2,6)	1 (10)
AdV + SARS-CoV-2	0	2 (5,2)	0
Parainfluenza 1 + Parainfluenza 2	0	1 (2,6)	0
SARS-CoV-2 + RSV + Influenza B	0	1 (2,6)	0
Influenza A + SARS-CoV-2	0	2 (5,2)	2 (20)
HMPV + SARS-CoV-2	0	1 (2,6)	0
HMPV + EV/RV	0	1 (2,6)	0
Influenza A + EV/RV + SARS-CoV-2	0	0	1 (10)
Influenza A + CoV 229E	0	0	1 (10)
AdV + BoV	0	0	1 (10)
Parainfluenza 3 + EV/RV	0	0	1 (10)
**Total**	**6**	**38**	**10**

## Data Availability

All epidemiological data are included in the main text.

## Supplementary Materials

The following supporting information can be downloaded at: https://www.mdpi.com/article/10.3390/v17081040/s1, Table S1: Detected respiratory viruses using the multiplex PCR assays.

## References

[1] Respiratory Infectious Diseases on the Rise Across WHO European Region. https://www.who.int/europe/news/item/15-12-2023-respiratory-infectious-diseases-on-the-rise-across-who-european-region.

[2] Trifonova I., Christova I., Madzharova I., Angelova S., Voleva S., Yordanova R., Tcherveniakova T., Krumova S., Korsun N. (2022). Clinical Significance and Role of Coinfections with Respiratory Pathogens among Individuals with Confirmed Severe Acute Respiratory Syndrome Coronavirus-2 Infection. Front. Public Health.

[3] Pavia G., Scarpa F., Ciccozzi A., Romano C., Branda F., Quirino A., Marascio N., Matera G., Sanna D., Ciccozzi M. (2024). Changing and Evolution of Influenza Virus: Is It a Trivial Flu?. Chemotherapy.

[4] Pavia G., Quirino A., Marascio N., Veneziano C., Longhini F., Bruni A., Garofalo E., Pantanella M., Manno M., Gigliotti S. (2024). Persistence of SARS-CoV-2 Infection and Viral *Intra*- and *Inter*- host Evolution in COVID-19 Hospitalized Patients. J. Med. Virol..

[5] Branda F., Pavia G., Ciccozzi A., Quirino A., Marascio N., Matera G., Romano C., Locci C., Azzena I., Pascale N. (2024). Zoonotic Paramyxoviruses: Evolution, Ecology, and Public Health Strategies in a Changing World. Viruses.

[6] De Marco C., Veneziano C., Massacci A., Pallocca M., Marascio N., Quirino A., Barreca G.S., Giancotti A., Gallo L., Lamberti A.G. (2022). Dynamics of Viral Infection and Evolution of SARS-CoV-2 Variants in the Calabria Area of Southern Italy. Front. Microbiol..

[7] Cui C., Timbrook T.T., Polacek C., Heins Z., Rosenthal N.A. (2024). Disease Burden and High-Risk Populations for Complications in Patients with Acute Respiratory Infections: A Scoping Review. Front. Med..

[8] Zhao P., Zhang Y., Wang J., Li Y., Wang Y., Gao Y., Zhao M., Zhao M., Tan H., Tie Y. (2024). Epidemiology of Respiratory Pathogens in Patients with Acute Respiratory Infections during the COVID-19 Pandemic and after Easing of COVID-19 Restrictions. Microbiol. Spectr..

[9] Alimi Y., Lim W.S., Lansbury L., Leonardi-Bee J., Nguyen-Van-Tam J.S. (2017). Systematic Review of Respiratory Viral Pathogens Identified in Adults with Community-Acquired Pneumonia in Europe. J. Clin. Virol..

[10] Msemburi W., Karlinsky A., Knutson V., Aleshin-Guendel S., Chatterji S., Wakefield J. (2023). The WHO Estimates of Excess Mortality Associated with the COVID-19 Pandemic. Nature.

[11] Jester B., Uyeki T.M., Jernigan D.B., Tumpey T.M. (2019). Historical and Clinical Aspects of the 1918 H1N1 Pandemic in the United States. Virology.

[12] Shi T., Denouel A., Tietjen A.K., Campbell I., Moran E., Li X., Campbell H., Demont C., Nyawanda B.O., Chu H.Y. (2020). Global Disease Burden Estimates of Respiratory Syncytial Virus-Associated Acute Respiratory Infection in Older Adults in 2015: A Systematic Review and Meta-Analysis. J. Infect. Dis..

[13] Li Y., Wang X., Blau D.M., Caballero M.T., Feikin D.R., Gill C.J., Madhi S.A., Omer S.B., Simões E.A.F., Campbell H. (2022). Global, Regional, and National Disease Burden Estimates of Acute Lower Respiratory Infections Due to Respiratory Syncytial Virus in Children Younger than 5 Years in 2019: A Systematic Analysis. Lancet Lond. Engl..

[14] Oh D.-Y., Buda S., Biere B., Reiche J., Schlosser F., Duwe S., Wedde M., von Kleist M., Mielke M., Wolff T. (2021). Trends in Respiratory Virus Circulation Following COVID-19-Targeted Nonpharmaceutical Interventions in Germany, January–September 2020: Analysis of National Surveillance Data. Lancet Reg. Health Eur..

[15] Lu Y., Chen Q., Ren S., Zhang Y., Yi L., Qian C., Shen J., Liu X., Jiang M., Wang B. (2024). Impact of COVID-19 Nonpharmaceutical Interventions on Respiratory Syncytial Virus Infections in Hospitalized Children. Influenza Other Respir. Viruses.

[16] Cohen R., Ashman M., Taha M.-K., Varon E., Angoulvant F., Levy C., Rybak A., Ouldali N., Guiso N., Grimprel E. (2021). Pediatric Infectious Disease Group (GPIP) Position Paper on the Immune Debt of the COVID-19 Pandemic in Childhood, How Can We Fill the Immunity Gap?. Infect. Dis. Now.

[17] Baker R.E., Park S.W., Yang W., Vecchi G.A., Metcalf C.J.E., Grenfell B.T. (2020). The Impact of COVID-19 Nonpharmaceutical Interventions on the Future Dynamics of Endemic Infections. Proc. Natl. Acad. Sci. USA.

[18] Zhu X., Ge Y., Wu T., Zhao K., Chen Y., Wu B., Zhu F., Zhu B., Cui L. (2020). Co-Infection with Respiratory Pathogens among COVID-2019 Cases. Virus Res..

[19] Carstens G., Kozanli E., Bulsink K., McDonald S., Elahi M., de Bakker J., Schipper M., van Gageldonk-Lafeber R., van den Hof S., Jan van Hoek A. (2025). Co-Infection Dynamics of SARS-CoV-2 and Respiratory Viruses in the 2022/2023 Respiratory Season in the Netherlands. J. Infect..

[20] Yan X., Li K., Lei Z., Luo J., Wang Q., Wei S. (2023). Prevalence and Associated Outcomes of Coinfection between SARS-CoV-2 and Influenza: A Systematic Review and Meta-Analysis. Int. J. Infect. Dis..

[21] Wong A., Barrero Guevara L.A., Goult E., Briga M., Kramer S.C., Kovacevic A., Opatowski L., Domenech de Cellès M. (2023). The Interactions of SARS-CoV-2 with Cocirculating Pathogens: Epidemiological Implications and Current Knowledge Gaps. PLoS Pathog..

[22] Clark T.W., Lindsley K., Wigmosta T.B., Bhagat A., Hemmert R.B., Uyei J., Timbrook T.T. (2023). Rapid Multiplex PCR for Respiratory Viruses Reduces Time to Result and Improves Clinical Care: Results of a Systematic Review and Meta-Analysis. J. Infect..

[23] The BioFire^®^ FilmArray^®^ Respiratory Panels (RP & RP2). https://www.biofiredx.com/products/the-filmarray-panels/rp-2-1-plus-panel/.

[24] QIAstat-Dx SARS-CoV-2. https://www.qiagen.com/ca/products/diagnostics-and-clinical-research/infectious-disease/qiastat-dx-syndromic-testing/qiastat-dx-ca.

[25] Brunstein J.D., Cline C.L., McKinney S., Thomas E. (2008). Evidence from Multiplex Molecular Assays for Complex Multipathogen Interactions in Acute Respiratory Infections. J. Clin. Microbiol..

[26] Peng D., Zhao D., Liu J., Wang X., Yang K., Xicheng H., Li Y., Wang F. (2009). Multipathogen Infections in Hospitalized Children with Acute Respiratory Infections. Virol. J..

[27] Chen A.P.-L., Chu I.Y.-H., Yeh M.-L., Chen Y.-Y., Lee C.-L., Lin H.-H., Chan Y.-J., Chen H.-P. (2021). Differentiating Impacts of Non-Pharmaceutical Interventions on Non-Coronavirus Disease-2019 Respiratory Viral Infections: Hospital-Based Retrospective Observational Study in Taiwan. Influenza Other Respir. Viruses.

[28] Ching N.S., Kotsanas D., Easton M.L., Francis M.J., Korman T.M., Buttery J.P. (2018). Respiratory Virus Detection and Co-Infection in Children and Adults in a Large Australian Hospital in 2009-2015. J. Paediatr. Child Health.

[29] Chong Y.M., Chan Y.F., Jamaluddin M.F.H., Hasan M.S., Pang Y.K., Ponnampalavanar S., Syed Omar S.F., Sam I.-C. (2022). Rhinovirus/Enterovirus Was the Most Common Respiratory Virus Detected in Adults with Severe Acute Respiratory Infections Pre-COVID-19 in Kuala Lumpur, Malaysia. PLoS ONE.

[30] Sim J.Y., Chen Y.-C., Hsu W.-Y., Chen W.-Y., Chou Y., Chow J.C., Lai Y.-C., Tang H.-J., Chen C.-C., Ho C.-H. (2022). Circulating Pediatric Respiratory Pathogens in Taiwan during 2020: Dynamic Change under Low COVID-19 Incidence. J. Microbiol. Immunol. Infect..

[31] Li W., Zhu Y., Lou J., Chen J., Xie X., Mao J. (2021). Rotavirus and Adenovirus Infections in Children during COVID-19 Outbreak in Hangzhou, China. Transl. Pediatr..

[32] Bardsley M., Morbey R.A., Hughes H.E., Beck C.R., Watson C.H., Zhao H., Ellis J., Smith G.E., Elliot A.J. (2023). Epidemiology of Respiratory Syncytial Virus in Children Younger than 5 Years in England during the COVID-19 Pandemic, Measured by Laboratory, Clinical, and Syndromic Surveillance: A Retrospective Observational Study. Lancet Infect. Dis..

[33] Li Y., Wang X., Cong B., Deng S., Feikin D.R., Nair H. (2022). Understanding the Potential Drivers for Respiratory Syncytial Virus Rebound During the Coronavirus Disease 2019 Pandemic. J. Infect. Dis..

[34] Orozco-Hernández J.P., Montoya-Martínez J.J., Pacheco-Gallego M.C., Céspedes-Roncancio M., Porras-Hurtado G.L. (2020). SARS-CoV-2 and Rhinovirus/Enterovirus Co-Infection in a Critically Ill Young Adult Patient in Colombia. Biomedica.

[35] Le Glass E., Hoang V.T., Boschi C., Ninove L., Zandotti C., Boutin A., Bremond V., Dubourg G., Ranque S., Lagier J.-C. (2021). Incidence and Outcome of Coinfections with SARS-CoV-2 and Rhinovirus. Viruses.

[36] Lade H., Kim J.-M., Chung Y., Han M., Mo E.-K., Kim J.-S. (2021). Comparative Evaluation of Allplex Respiratory Panels 1, 2, 3, and BioFire FilmArray Respiratory Panel for the Detection of Respiratory Infections. Diagnostics.

[37] Livingstone R., Lin H., Brendish N.J., Poole S., Tanner A.R., Borca F., Smith T., Stammers M., Clark T.W. (2022). Routine Molecular Point-of-Care Testing for SARS-CoV-2 Reduces Hospital-Acquired COVID-19. J. Infect..

[38] Cheng Y., Ma J., Wang H., Wang X., Hu Z., Li H., Zhang H., Liu X. (2021). Co-Infection of Influenza A Virus and SARS-CoV-2: A Retrospective Cohort Study. J. Med. Virol..

[39] Halfmann P.J., Nakajima N., Sato Y., Takahashi K., Accola M., Chiba S., Fan S., Neumann G., Rehrauer W., Suzuki T. (2022). SARS-CoV-2 Interference of Influenza Virus Replication in Syrian Hamsters. J. Infect. Dis..

[40] Oishi K., Horiuchi S., Minkoff J.M., tenOever B.R. (2022). The Host Response to Influenza A Virus Interferes with SARS-CoV-2 Replication during Coinfection. J. Virol..

[41] Dee K., Schultz V., Haney J., Bissett L.A., Magill C., Murcia P.R. (2023). Influenza A and Respiratory Syncytial Virus Trigger a Cellular Response That Blocks Severe Acute Respiratory Syndrome Virus 2 Infection in the Respiratory Tract. J. Infect. Dis..

[42] Berry G.J., Zhen W., Smith E., Manji R., Silbert S., Lima A., Harington A., McKinley K., Kensinger B., Neff C. (2022). Multicenter Evaluation of the BioFire Respiratory Panel 2.1 (RP2.1) for Detection of SARS-CoV-2 in Nasopharyngeal Swab Samples. J. Clin. Microbiol..

